# The Diseases of Females, Including Those of Pregnancy and Childbed

**Published:** 1850-06

**Authors:** 


Art. VI.—*The Diseases of Females, including those of Pregnancy and Childbed.* By Fleetwood Churchhill, M. D., author of "The Theory and Practice of Midwifery," and "Diseases of Infants," &c. A new American edition, revised by the author. With the notes of Robert M. Huston, M. D., Professor of Materia Medica and General Therapeutics in the Jefferson Medical College of Philadelphia, &c. Philadelphia: Lea & Blanchard. 1850.

This is the fifth American edition of Dr. Churchill's work on the "Diseases of Females," a fact that speaks expressively of the value of the book. In the present edition, Dr. Churchill says: "The valuable notes of my friend Dr. Huston have been carefully retained, and I have added considerably both to the text and notes, so that I trust it will be found an improvement upon its predecessors."

Dr. Churchill's distinguishing traits as an author are cautious, but copious description of disease, clearness in pointing to the means of diagnosis, and fullness in his therapeutical means. These traits have enabled Dr. Churchill to command the approbation of the medical profession in Great Britain and America. We know of no author who deserves that approbation, on "the diseases of females," to the same extent that Dr. Churchill does. His, indeed, is the only thorough treatise, we know of, on the subject, and it may be commended to practitioners and students as a masterpiece in its particular department.

The former editions of this work have been commended strongly in this Journal, and they have won their way to an extended, and a well deserved popularity. This fifth edition, before us, is well calculated to maintain Dr. Churchill's high reputation. It was revised and enlarged by the author, for his American publishers, and it seems to us, that there is scarcely any species of desirable information on its subjects, that may not be found in this work.

The American editor, too, has discharged his duty towards this fifth edition. We commend the work as one that should be found in the hands of every practitioner of medicine, and of every student, who has a real desire to adorn a noble profession, by practical talents.

The admirers of Dr. Churchill are indebted to Lea & Blanchard for the very creditable manner in which they have published this book.

Churchill on "The Diseases of Females," may be found at the bookstore of Beckwith & Morton.

